# Self-Reported Reasons Preventing US Adults From Walking to Places Within 10 Minutes of Home

**DOI:** 10.5888/pcd22.240394

**Published:** 2025-06-19

**Authors:** Hatidza Zaganjor, Tiffany J. Chen, Miriam E. Van Dyke, Graycie W. Soto, Geoffrey P. Whitfield, Akimi Smith, Heather M. Devlin, Katherine Irani, Ken Rose, Jennifer L. Matjasko

**Affiliations:** 1 Division of Nutrition, Physical Activity, and Obesity; National Center for Chronic Disease Prevention and Health Promotion; Centers for Disease Control and Prevention, Atlanta, Georgia; 2 Office on Smoking and Health; National Center for Chronic Disease Prevention and Health Promotion, Centers for Disease Control and Prevention, Atlanta, Georgia

## Abstract

**Introduction:**

Increasing walking for transportation is a strategy to integrate physical activity into daily life. We examined reported environmental, access, and individual reasons for not walking to places near home among US adults, by sociodemographic characteristics and geographic location.

**Methods:**

We used data from the 2022 SummerStyles survey on 3,967 US adults aged 18 years or older. We calculated prevalence of reporting 11 selected reasons for not walking to places within 10 minutes of home, overall and by sex, race or ethnicity, age, education, income, US census region, and metropolitan residence (an area with at least 1 urban area of ≥50,000 inhabitants) versus nonmetropolitan residence. We used Bonferroni-corrected pairwise comparisons and orthogonal polynomial contrasts (ordered groups) to compare prevalence by subgroup.

**Results:**

Overall, 79.0% of respondents identified at least 1 reason for not walking to places near home (within 10 minutes). Commonly reported reasons were hot and humid conditions (36.0%), no places to walk within 10 minutes (24.9%), a preference for driving (22.1%), and inconvenience (21.5%). The reasons varied significantly across sociodemographic and geographic subgroups. The prevalence of reporting none of the listed reasons was higher among males than females, higher among non-Hispanic Black and non-Hispanic Asian adults than non-Hispanic White adults, and higher among adults from the Northeast versus the South.

**Conclusion:**

Eight of 10 US adults reported at least 1 environmental, access, or individual reason for not walking to places near home. Designing communities to make walking for transportation more accessible, convenient, and desirable may help address the leading reasons reported, which may support adults in adding more physical activity to their daily lives.

**Summary**

**What is already known on this topic?**

Walking is the most common form of transportation-related physical activity in the US.

**What is added by this report?**

Data from the 2022 SummerStyles survey show that 8 of 10 adults reported at least 1 reason for not walking to places within a 10-minute walk from their homes. Prevalence of reported reasons varied by sociodemographic characteristics and geographic location.

**What are the implications for public health practice?**

Designing communities to make walking near home more accessible, convenient, and desirable can help adults integrate physical activity into daily life.

## Introduction

Transportation-related physical activity (ie, active transportation) is a way Americans can engage in aerobic physical activity. Active transportation is defined as trips from 1 destination to another through physically active means such as walking, bicycling, or using other means of human-powered mobility (1). Among active transportation modes, walking is the most common in the US (2). Shifts to active transportation modes from motorized vehicles can address negative health effects associated with physical inactivity, air pollution, greenhouse gases, and traffic injuries (3). Increasing walkability, for example, is supported by the Center for Disease Control and Prevention (CDC’s) Active People, Healthy Nation Initiative as a way to integrate physical activity into daily life (4).

Despite the health benefits of active transportation, for individuals and the population overall, transportation walking is uncommon in the US and accounts for only about 12% of all trips (5). According to data from the US Department of Transportation’s National Household Travel Survey, about a quarter of trips of 1 mile or less are walked (2). This suggests that many short-distance trips made via motorized transportation modes could be opportunities for walking. To better plan interventions to increase transportation walking, a better understanding is needed of the reasons people do not walk to near-home destinations.

The Surgeon General’s Call to Action to Promote Walking and Walkable Communities identifies several barriers to overall walking for different populations. These include a lack of time, safety, community design, and personal ability (6). However, current national data related to walking to nearby destinations are limited. One study assessed barriers to walking in response to neighborhood environments and found, compared to people who do not walk for transportation, those who walk for transportation were 1.3 times more likely to perceive vehicle speeding as a safety barrier in their neighborhood (7). Another study identified self-reported barriers related to traffic, crime, and animals (8). Traffic, which was the most reported of the 3 barriers, was reported by 23.4% of adults with no differences by race or ethnicity and was most prevalent among adults in the South. Crime (12.4%) and animals (10.5%) were less commonly reported.

The decision to engage in transportation walking may be influenced by a variety of factors, as described in a multi-layered model of active living (9). This model posits that behavior is influenced by wide-ranging factors at multiple levels, such as intrapersonal (eg, psychological, biological), perceived environments (eg, safety, convenience, aesthetics), and behavior settings (eg, traffic, walking facilities, parks). A fuller understanding of reasons for not walking to places near home and how they vary across populations can inform strategies to increase physical activity through transportation walking. Our study assessed the prevalence of reporting selected reasons for not walking to places near home among US adults, overall and by sociodemographic characteristics and geographic location.

## Methods

### Study population

We used data from the 2022 SummerStyle*s* wave of Porter Novelli’s ConsumerStyles survey (10). Participants for this consumer survey are recruited from an online database of panel members via the Ipsos KnowledgePanel (10), a representative sample of the noninstitutionalized US population. Panel members are randomly recruited by using probability-based sampling by home address. The panel is continuously replenished and maintains approximately 60,000 members. The 2022 SummerStyles survey was fielded from May 31 through July 6, 2022. The survey was sent to 5,990 households that completed the SpringStyles survey, the first wave of the 2022 ConsumerStyles survey. Adults who completed the survey received cash-equivalent reward points worth approximately $5. A total of 4,156 members completed the 2022 SummerStyles survey (response rate, 69.3%). Data were weighted to match the 2021 US Current Population Survey proportions for sex, by age, household income, race and ethnicity, household size, education, census region, metropolitan status, and parental status of children aged 11 to 17 years (10). A total of 4.5% (n = 189) of respondents were excluded because of missing data on reasons for not walking or for reporting being unable to walk. Our final analytic sample (N = 3,967) contained significantly more males, adults with higher education levels, and adults with higher incomes compared with those who were excluded from the analysis.

### Reasons for not walking

Respondents were asked, “Which of the following prevent you from regularly walking to places within a 10-minute walk of where you live?” They were asked to select from a list of 11 predetermined reasons. We drew upon the ecological model of active living (9) to sort our 11 reasons into 3 categories: environmental, access, and individual (9). The environmental category comprised reasons related to the perceived natural environment and general surroundings: hot or humid conditions, feeling unsafe for any reason, cold or icy conditions, and an unpleasant or unhealthy environment. The access category listed reasons related to the presence of routes or destinations. Choices in this category were “there are no places within a 10-minute walk of where I live,” “sidewalks are missing or poorly maintained,” and “crosswalks are missing or too far apart.” Choices in the individual category were intrapersonal preferences and perceived fitness levels: “prefer driving or being driven,” “inconvenient (eg, too far, takes too long, unfamiliar),” “my physical abilities or fitness,” and “do not like walking.” If none of the response options applied, respondents could select “none of the above.”

### Respondent characteristics

Respondents self-reported their age (18–34, 35–49, 50–64, ≥ 65 years), sex (female, male), race or ethnicity (non-Hispanic Asian, non-Hispanic Black, Hispanic or Latino/a, non-Hispanic multiracial or another race, or non-Hispanic White), education level (high school diploma or less, some college, bachelor’s degree or higher), annual household income (<$50,000, $50,000–$99,999, ≥$100,000), US census region (Northeast, Midwest, South, West), and its census status (nonmetropolitan, metropolitan). Metropolitan was defined as an urban area of 50,000 residents or more (11).

### Statistical analyses

We calculated weighted prevalence and 95% CIs for respondent reasons for not walking near home, overall and by respondent characteristics, and used Bonferroni-corrected pairwise *t* tests to assess differences between subgroups and polynomial orthogonal contrasts to assess linear trends across ordered subgroups. We set significance at *P* < .05 and accounted for weighting in SUDAAN v11.0.1 (RTI International). Because our study involved the examination of precollected data licensed from Porter Novelli Public Services, CDC’s institutional review board determined it to be exempt.

## Results

### Sample characteristics

Most of the weighted sample of adults (70.6%) were aged 35 years or older, were non-Hispanic White (63.0%), had at least some college education (63.5%), had an annual household income of $50,000 or more (71.6%), and lived in a metropolitan area (87.0%) (Table 1).

**Table 1:** Respondent Characteristics, 2022 Summer Styles Survey (N = 3,967)^a^

Characteristic	n	Unweighted %	Weighted % (95% CI)^b^
**Age, y**			
18–34	576	14.5	29.3 (27.4–31.4)
35–49	1,105	27.9	23.7 (22.2–25.3)
50–64	1,126	28.4	24.8 (23.4–26.4)
≥65	1,160	29.2	22.1 (20.8–23.4)
**Sex**			
Male	1,996	50.3	49.4 (47.5–51.3)
Female	1,971	49.7	50.6 (48.7–52.5)
**Race or ethnicity**			
Hispanic or Latino/a	443	11.2	16.9 (15.3–18.6)
Non-Hispanic Asian	164	4.1	5.9 (5.0–7.0)
Non-Hispanic Black	358	9.0	11.7 (10.5–13.1)
Non-Hispanic White	2,859	72.1	63.0 (61.0–64.9)
Non-Hispanic multiracial or another race	143	3.6	2.6 (2.0–3.2)
**Education**			
High school diploma or less	1,155	29.1	36.6 (34.7–38.5)
Some college	1,113	28.1	27.3 (25.6–28.9)
Bachelor's degree or higher	1,699	42.8	36.2 (34.4–37.9)
**Income, $**			
<50,000	937	23.6	28.4 (26.6–30.2)
50,000–99,999	1,186	29.9	30.1 (28.4–31.9)
≥100,000	1,844	46.5	41.5 (39.7–43.4)
**Region**			
Northeast	701	17.7	17.2 (15.8–18.6)
Midwest	893	22.5	20.9 (19.4–22.4)
South	1,419	35.8	37.9 (36.1–39.8)
West	954	24.0	24.1 (22.5–25.7)
**Metropolitan statistical area status**			
Nonmetropolitan	497	12.5	13.0 (11.8–14.3)
Metropolitan	3,470	87.5	87.0 (85.7–88.2)

a We excluded 189 adults because of missing factors preventing walking or because participants responded they were unable to walk.

b Weighted to the total US population as estimated by the 2021 US Current Population Survey proportions using 8 factors (sex by age, household income, race or ethnicity, household size, education, census region, metropolitan status, and parental status of children aged 11 to 17 years).

### Prevalence of reasons for not walking to places near home, by type

Most adults (79.0%) reported at least 1 reason for not walking to places within a 10-minute walk of their homes, with only 21% reporting “none of the above” (Figure).

**Figure:**
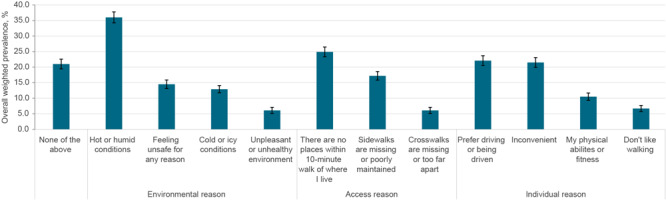
Weighted prevalence of reasons reported by adults for not walking to places near home, by type, 2022 SummerStyles survey, United States (9). Error bars indicate 95% CIs. Respondents were asked, “Which of the following prevent you from regularly walking to places within a 10-min walk of where you live?

**Table:** 

Reason	Overall weighted prevalence, % (95% CI)
Environmental reasons	
Hot or humid conditions	36.0 (34.2–37.8)
Feeling unsafe for any reason	14.5 (13.2–16.0)
Cold or icy conditions	12.9 (11.8–14.1)
Unpleasant or unhealthy environment	6.1 (5.2–7.2)
Access reasons	
There are no places within 10-minute walk of where I live	24.9 (23.4–26.5)
Sidewalks missing or poorly maintained	17.2 (15.8–18.6)
Crosswalks missing or too far apart	6.1 (5.2–7.1)
Individual reasons	
Prefer driving or being driven	22.1 (20.5–23.8)
Inconvenient	21.5 (20.0–23.1)
My physical abilities or fitness	10.5 (9.4–11.6)
Don’t like walking	6.7 (5.8–7.8)
None of the above	21.0 (19.5–22.6)

Among environmental reasons, 36.0% (95% CI, 34.2%–37.8%) of adults reported hot or humid conditions as a reason more than twice as frequently as the next most common reason, feeling unsafe (14.5%; 95% CI, 13.2%–16.0%). Cold or icy conditions (12.9%; 95% CI, 11.8%– 14.1%) and an unpleasant or unhealthy environment (6.1%; 95% CI, 5.2%–7.2%) were less commonly reported. All environmental reasons varied by at least 1 sociodemographic or geographic characteristic. For example, more females (39.0%) reported hot or humid conditions compared with males (32.9%). Additionally, more non-Hispanic White (36.8%) and Hispanic or Latino/a (37.8%) adults reported hot and humid conditions compared with non-Hispanic Asian adults (23.2%). Finally, more adults living in the South (46.2%) reported hot and humid conditions compared with those living in the Northeast (29.5%), Midwest (31.5%), or the West (28.3%) (Table 2).

**Table 2:** Prevalence of Environmental Reasons for Not Walking to Places Near Home, by Sociodemographic and Geographic Characteristics, 2022

Characteristic	Environmental reasons, % (95% CI)			
	Hot or humid conditions	Feeling unsafe for any reason	Cold or icy conditions	Unpleasant or unhealthy environment (e.g., trash, noise, pollution)
**Overall**	36.0 (34.2–37.8)	14.5 (13.2–16.0)	12.9 (11.8–14.1)	6.1 (5.2–7.2)
**Age, y**				
18–34	39.1 (34.9–43.4)^a^	18.5 (15.3­22.2)^a^^,^ b	a9.6 (7.5–12.2)^b^	8.3 (6.2–11.1)^a^^,^ b
35–49	35.0 (31.6–38.5)	14.4 (11.9–17.2)	12.1 (9.9–14.6)	6.4 (4.8–8.6)
50–64	36.5 (33.3–39.7)	13.6 (11.4–16.2)	15.0 (12.8–17.5)^c^	5.3 (3.9–7.2)
≥65	32.4 (29.6–35.4)	10.3 (8.5–12.4)^c^	15.9 (13.8–18.2)^c^	3.8 (2.7–5.2)^c^
**Sex**				
Male	32.9 (30.4–35.4)^b^	10.2 (8.6–12.0)^b^	12.5 (10.9–14.2)	5.3 (4.2–6.7)
Female	39.0 (36.4–41.7)^c^	18.7 (16.6–21.0)^c^	13.4 (11.8–15.2)	6.9 (5.6–8.6)
**Race or ethnicity**				
Hispanic or Latino/a	37.8 (32.6–43.3)^b^	21.8 (17.5–26.9)^b^	8.9 (6.4–12.2)^b^	7.8 (5.3–11.4)
Non-Hispanic Asian	23.2 (16.7–31.3)^c^	13.7 (8.7–20.9)	12.1 (7.5–18.8)	12.2 (7.5–19.3)
Non-Hispanic Black	35.7 (30.2–41.7)	15.6 (11.9–20.3)	9.7 (6.8–13.7)	5.3 (3.0–9.3)
Non-Hispanic White	36.8 (34.7–38.9)^b^	12.1 (10.7–13.7)^c^	14.7 (13.4–16.3)^c^	5.2 (4.3–6.4)
Non-Hispanic multiracial or another race	35.1 (25.4–46.2)	21.2 (13.2–32.4)	11.0 (6.2–18.8)	—d
**Education**				
High school diploma or less	36.6 (33.4–40.0)	13.9 (11.6–16.6)	11.0 (9.2–13.1)^ a,b^	6.5 (4.8–8.6)
Some college	38.7 (35.4–42.2)^b^	17.0 (14.4–19.9)	13.0 (10.9–15.4)	6.0 (4.5–7.9)
Bachelor's degree or higher	33.2 (30.6–35.9)^c^	13.2 (11.3–15.4)	14.8 (13.0–16.9)^c^	5.9 (4.6–7.5)
**Income, $**				
<50,000	41.8 (38.1–45.6)^a^^,^ b	19.3 (16.4–22.5)^a^^,^ b	12.9 (10.8–15.4)	7.8 (6.0–10.2)
50,000–99,999	35.7 (32.5–39.1)	14.2 (11.9–16.9)^c^	12.4 (10.5–14.7)	5.3 (3.9–7.2)
≥100,000	32.2 (29.7–34.8)^c^	11.5 (9.7–13.5)^c^	13.3 (11.6–15.1)	5.5 (4.2–7.1)
**Region**				
Northeast	29.5 (25.7–33.7)^b^	12.2 (9.6–15.5)	20.3 (17.1–23.9)^b^	6.6 (4.5–9.7)
Midwest	31.5 (28.0–35.3)^b^	14.6 (11.8–17.8)	20.3 (17.5–23.5)^b^	4.2 (2.9–6.2)^b^
South	46.2 (43.1–49.4)^c^	14.4 (12.2–17.0)	7.8 (6.4–9.5)^c^	4.8 (3.5–6.4)^b^
West	28.3 (24.8–32.0)^b^	16.1 (13.4–19.3)	9.3 (7.4–11.7)^c^	9.5 (7.3–12.2)^c^
**Metropolitan statistical area status**				
Nonmetropolitan	31.8 (27.1–36.8)	12.2 (9.1–16.2)	14.7 (11.6–18.6)	3.9 (2.3–6.6)^b^
Metropolitan	36.6 (34.7–38.6)	14.8 (13.4–16.4)	12.7 (11.5–13.9)	6.4 (5.4–7.6)^c^

a Ordinal subgroups with superscript a have a significant linear trend (*P *< .05).

b Within a characteristic, subgroup values with superscript letter b are significantly different from values with superscript letter c (*P *< .05, Bonferroni corrected for multiple pairwise comparisons).

c Within a characteristic, subgroup values with superscript letter c are significantly different from values with superscript letter b (*P* < .05, Bonferroni corrected for multiple pairwise comparisons).

d Denotes a subgroup with a suppressed unstable estimate.

Among access reasons, 24.9% (95% CI, 23.4%–26.5%) reported having no places to walk within 10 minutes of home, 17.2% (95% CI, 15.8%–18.6%) reported sidewalks missing or poorly maintained, and 6.1% (95% CI, 5.2%–7.1%) reported crosswalks missing or too far apart. Prevalence of all access reasons differed across multiple sociodemographic or geographic characteristics. For example, the prevalence of reporting no places within 10 minutes was higher among non-Hispanic White adults (28.7%) than non-Hispanic Black (17.2%), Hispanic or Latino/a (20.2%), or non-Hispanic Asian adults (13.1%). Additionally, more adults with at least a bachelor’s degree (27.5%) reported this reason compared with adults with a high school diploma or less (21.7%). Adults with incomes between $50,000 and $99,999 (25.9%) and incomes of $100,000 or more (27.7%) were also more likely to report no places within 10 minutes as a reason for not walking near home than were adults with incomes less than $50,000 (19.7%). This reason also varied by geographic location. More adults in the South (27.5%) reported this reason compared with those living in the West (21.1%), and more adults living in nonmetropolitan areas (31.5%) reported this reason than adults living in metropolitan areas (23.9%). (Table 3).

**Table 3:** Prevalence of Access Reasons for Not Walking to Places Near Home by Sociodemographic and Geographic Characteristics, 2022

Characteristic	Access reasons, % (95% CI)		
	There are no places within a 10-minute walk of where I live	Sidewalks are missing or poorly maintained	Crosswalks are missing or too far apart
**Overall**	24.9 (23.4–26.5)	17.2 (15.8–18.6)	6.1 (5.2–7.1)
**Age, y**			
18–34	22.4 (19.0–26.3)	21.3 (18.0–25.0)^ a,b^	8.6 (6.6–11.4)^ a,b^
35–49	26.0 (23.0–29.1)	17.9 (15.3–20.8)	7.3 (5.6–9.6)^b^
50–64	26.1 (23.4–29.0)	14.8 (12.7–17.3)^c^	4.9 (3.7–6.6)^b^
≥65	25.6 (23.1–28.3)	13.6 (11.7–15.8)^c^	2.6 (1.8–3.7)^c^
**Sex**			
Female	26.3 (24.1–28.6)	19.5 (17.4–21.7)^b^	7.1 (5.8–8.8)^b^
Male	23.5 (21.4–25.7)	14.8 (13.0–16.8)^c^	5.0 (4.0–6.4)^c^
**Race or ethnicity**			
Hispanic or Latino/a	20.2 (16.1–24.9)^b^	18.1 (14.2–22.8)	7.8 (5.3–11.5)
Non-Hispanic Asian	13.1 (8.0–20.6)^b^	10.4 (6.3–16.7)	—d
Non-Hispanic Black	17.2 (13.2–22.1)^b^	14.7 (10.9–19.6)	4.6 (2.7–7.9)
Non-Hispanic White	28.7 (26.8–30.6)^c^	18.0 (16.4–19.8)	6.0 (5.0–7.2)
Non-Hispanic multiracial or another race	25.0 (16.9–35.3)	16.6 (10.8–24.7)	—d
**Education**			
High school diploma or less	21.7 (19.1–24.5)^ a,b^	14.4 (12.2–17.0)^ a,b^	3.3 (2.3–4.7)^ a,b^
Some college	25.7 (22.9–28.8)	19.2 (16.6–22.2)^c^	8.1 (6.1–10.6)^c^
Bachelor's degree or higher	27.5 (25.1–30.1)^c^	18.4 (16.3–20.8)^c^	7.5 (6.0–9.2)^c^
**Income, $**			
<50,000	19.7 (16.9–22.8)^ a b^	18.8 (15.9–22.0)	5.3 (3.8–7.5)
50,000–99,999	25.9 (23.1–28.9)^c^	16.9 (14.5–19.6)	5.7 (4.3–7.6)
≥100,000	27.7 (25.4–30.2)^c^	16.3 (14.3–18.4)	6.9 (5.6–8.5)
**Region**			
Northeast	22.8 (19.4–26.5)	16.6 (13.6–20.0)	6.8 (4.7–9.7)
Midwest	26.3 (23.0–29.8)	17.4 (14.6–20.7)	5.5 (3.8–7.9)
South	27.5 (24.9–30.3)^b^	20.0 (17.6–22.6)^b^	6.0 (4.7–7.7)
West	21.1 (18.1–24.4)^c^	13.0 (10.6–15.8)^c^	6.2 (4.5–8.5)
**Metropolitan statistical area status**			
Nonmetropolitan	31.5 (27.1–36.3)^b^	18.0 (14.5–22.2)	3.7 (2.2–6.1)^b^
Metropolitan	23.9 (22.3–25.6)^c^	17.0 (15.6–18.6)	6.5 (5.5–7.6)^c^

a Ordinal subgroups with superscript a have a significant linear trend (*P* < .05).

b Within a characteristic, subgroup values with superscript letter b are significantly different from values with superscript letter (*P* < .05, Bonferronci corrected for multiple pairwise comparisons).

c Within a characteristic, subgroup values with superscript letter c are significantly different from values with superscript letter b (*P* < .05, Bonferroni corrected for multiple pairwise comparisons).

d Denotes a subgroup with a suppressed unstable estimate.

Among individual reasons, respondents reported preferring driving or being driven (22.1%; 95% CI, 20.5%–23.8%) and inconvenience (21.5%; 95% CI, 20.0%–23.1%) more than twice as often as the next most frequently reported reasons, “my physical abilities or fitness” (10.5%; 95% CI, 9.4%–11.6%) and “do not like walking” (6.7%; 95% CI, 5.8%–7.8%). All individual reasons differed across multiple sociodemographic or geographic characteristics. For example, the prevalence of preferring driving or being driven decreased in older age groups. More females (23.9%) reported this reason than males (20.3%), but that number decreased as education level increased. In addition, more adults living in metropolitan areas (22.8%) reported preferring driving or being driven compared with adults in nonmetropolitan areas (17.7%). (Table 4).

**Table 4:** Prevalence of Individual Reasons for Not Walking to Places Near Home by Sociodemographic and Geographic Characteristics, 2022

Characteristic	Individual reasons, % (95% CI)			
	Prefer driving or being driven	Inconvenient (eg, too far, takes too long, unfamiliar)	My physical abilities or fitness	Do not like walking
**Overall**	22.1 (20.5–23.8)	21.5 (20.0–23.1)	10.5 (9.4–11.6)	6.7 (5.8–7.8)
**Age, y **				
18–34	28.3 (24.4–32.5)^ a,b^	29.2 (25.4–33.3)^ a,b^	4.1 (2.7–6.4)^ a,b^	9.2 (6.9–12.3)^ a,b^
35–49	21.9 (19.1–25.0)	21.4 (18.7–24.4)^c^^,^ d	6.3 (4.7–8.4)^b^	6.2 (4.7–8.2)
50–64	19.2 (16.7–22.1)^c^	18.6 (16.2–21.4)^c^	12.1 (10.0–14.6)^c^^,^ d	4.9 (3.7–6.6)^c^
≥65	17.4 (15.2–19.9)^c^	14.5 (12.6–16.7)^c^^,^ e	21.5 (19.0–24.2)^c^^,^ e	6.1 (4.7–7.8)
**Sex**				
Male	20.3 (18.2–22.6)^b^	20.8 (18.7–23.1)	9.3 (7.9–10.8)^b^	7.5 (6.1–9.1)
Female	23.9 (21.6–26.4)^c^	22.1 (19.9–24.5)	11.7 (10.1–13.4)^c^	6.0 (4.8–7.6)
**Race or ethnicity**				
Hispanic or Latino/a	25.2 (20.6–30.3)	22.3 (17.9–27.3)	11.2 (8.3–14.9)	7.2 (4.8–10.8)
Non-Hispanic Asian	22.0 (15.2–30.8)	20.7 (14.0–29.5)	—f	—f
Non-Hispanic Black	18.6 (14.5–23.7)	15.1 (11.3–19.9)^b^	8.3 (5.7–11.9)	5.3 (3.1–8.9)
Non-Hispanic White	22.3 (20.4–24.2)	22.8 (21.0–24.7)^c^	11.5 (10.2–12.9)	7.0 (5.9–8.4)
Non-Hispanic multiracial or another race	14.8 (9.3–22.7)	15.1 (9.7–22.9)	—f	—f
**Education**				
High school diploma or less	25.6 (22.7–28.8)^ a,b ^	16.9 (14.4–19.7)^ a,b^	13.9 (11.8–16.3)^ a,b^	8.8 (6.9–11.2)^ a,b^
Some college	22.9 (20.0–26.0)^b^	23.4 (20.5–26.5)^c^	12.1 (10.2–14.3)^b^	7.4 (5.8–9.6)^b^
Bachelor's degree or higher	18.0 (15.8–20.4)^c^	24.7 (22.3–27.3)^c^	5.8 (4.8–7.0)^c^	4.1 (3.1–5.4)^c^
**Income, $**				
<50,000	23.5 (20.4–27.0)	18.9 (16.0–22.2)^a^	16.5 (14.1–19.2)^ a,b^	8.5 (6.6–10.9)^ a,b^
50,000–99,999	23.8 (20.9–27.0)	20.9 (18.2–23.8)	10.6 (8.8–12.7)^cd^	7.0 (5.3–9.2)
≥100,000	19.9 (17.7–22.4)	23.7 (21.4–26.2)	6.3 (5.1–7.7)^ce^	5.4 (4.1–6.9)^c^
**Region**				
Northeast	19.4 (15.9–23.3)	18.9 (15.5–22.9)^b^	10.7 (8.5–13.4)	3.9 (2.5–5.9)^b^
Midwest	22.9 (19.7–26.5)	22.3 (19.2–25.8)	10.3 (8.2–12.8)	7.9 (5.8–10.6)^c^
South	22.6 (20.0–25.5)	19.1 (16.8–21.6)^b^	11.1 (9.4–13.1)	7.7 (6.1–9.6)^c^
West	22.6 (19.4–26.2)	26.4 (22.9–30.1)^c^	9.5 (7.5–11.8)	6.4 (4.6–8.7)
**Metropolitan statistical area status**				
Nonmetropolitan	17.7 (14.0–22.1)^b^	19.8 (15.9–24.4)	13.6 (10.5–17.4)	7.1 (4.7–10.8)
Metropolitan	22.8 (21.1–24.6)^c^	21.7 (20.1–23.5)	10.0 (8.9–11.2)	6.7 (5.7–7.8)

a Ordinal subgroups with superscript a have a significant linear trend (*P* < .05).

b Within a characteristic, subgroup values with superscript letter b are significantly different from values with superscript letter c (*P* < .05, Bonferroni corrected for multiple pairwise comparisons).

c Within a characteristic, subgroup values with superscript letter c are significantly different from values with superscript letter b (*P *< .05, Bonferroni corrected for multiple pairwise comparisons).

d Within a characteristic, subgroup values with superscript letter d are significantly different from values with superscript letter e (*P *< .05, Bonferroni corrected for multiple pairwise comparisons).

e Within a characteristic, subgroup values with superscript letter e are significantly different from values with superscript letter d (P < .05, Bonferroni corrected for multiple pairwise comparisons).

f Denotes a subgroup with a suppressed unstable estimate.

Among adults who indicated “none of the above” (no listed reasons) (21.0%; 95% CI, 19.5%–22.6%), more were males (25.8%) than females (16.3%), non-Hispanic Black (28.3%) or non-Hispanic Asian (30.8%) than non-Hispanic White (18.5%), and more lived in the Northeast (24.7%) than in the South (18.6%) (Table 5).

**Table 5:** Prevalence of No Listed Reasons for Not Walking to Places Near Home by Sociodemographic and Geographic Characteristics, 2022

Characteristic	None of the above, % (95% CI)^a^
**Overall**	21.0 (19.5–22.6)
**Age, y**	
18–34	20.9 (17.5–24.8)
35–49	21.5 (18.5–24.7)
50–64	20.2 (17.7–22.9)
≥65	21.3 (19.0–23.9)
**Sex**	
Male	25.8 (23.4–28.2)^b^
Female	16.3 (14.5–18.3)^c^
**Race or ethnicity**	
Hispanic or Latino/a	21.1 (17.0–26.0)
Non-Hispanic Asian	30.8 (23.3–39.5)^b^
Non-Hispanic Black	28.3 (23.1–34.2)^b^
Non-Hispanic White	18.5 (16.9–20.2)^c^
Non-Hispanic multiracial or another race	23.2 (14.2–35.6)
**Education**	
High school diploma or less	22.0 (19.3–25.0)
Some college	19.0 (16.3–22.0)
Bachelor's degree or higher	21.4 (19.2–23.8)
**Income, $**	
<50,000	19.0 (16.2–22.3)
50,000–99,999	20.4 (17.7–23.4)
≥100,000	22.7 (20.4–25.1)
**Region**	
Northeast	24.7 (21.2–28.7)^b^
Midwest	18.6 (15.6–21.9)
South	18.6 (16.2–21.2)^c^
West	24.1 (20.8–27.6)
**Metropolitan statistical area status**	
Nonmetropolitan	17.7 (13.9–22.3)
Metropolitan	21.4 (19.8–23.2)

a None of the listed reasons reported.

b Within a characteristic, subgroup values with superscript letter (b) are significantly different from values with superscript letter c (*P* < .05, Bonferroni corrected for multiple pairwise comparisons).

c Within a characteristic, subgroup values with superscript letter (c) are significantly different from values with superscript letter b (*P* < .05, Bonferroni corrected for multiple pairwise comparisons).

## Discussion

Almost 8 of 10 US adults in our study reported environmental, access, or individual reasons for not walking to places near home (≤10 min walk). The prevalence of reasons varied by sociodemographic characteristics and geographic location. For example, more females reported environmental, access, and individual reasons than males, and more non-Hispanic White adults reported reasons across all 3 categories (environmental, access, and individual) compared with adults of other races or ethnicities. Some commonly reported reasons may be addressed through multi-layered interventions to support active transportation (12). For example, interventions can improve the variety of destinations, reduce distances to destinations, increase street connectivity, and strengthen safety; these elements can make walking an attractive and safer transportation option (13).

Our study includes several findings with implications for interventions to increase near-home transportation walking. Most notably, hot and humid conditions were a commonly reported reason for not walking to places near home, especially among adults living in the South compared with any other US region. Heat has become a growing public health concern (14). Although our study was fielded in the summer months of 2022 when many regions experienced periods of high temperatures, physical activity is highest in the summer season (15). Conversely, cold or icy conditions were selected as reasons by only 12.9% of respondents, with a greater proportion of those living in the Northeast and Midwest citing this reason compared with those in the South and West. This suggests that seasonality may partially explain some of our results. However, recall errors may be less of a concern when it comes to heat and humidity given that the survey was administered during the summer when physical activity behaviors are more likely (16), a potential strength of our study. Interventions to address or reduce hot and humid conditions may support walking. Objective measures of outdoor physical activity in Tennessee showed increased counts of people walking, running, or bicycling as temperatures increased, but counts peaked at 84 degrees Fahrenheit and decreased for every 1 degree above that (17). Interventions such as increasing tree canopy and green space can reduce air temperature and improve air quality (18). Such changes can reduce heat stress and improve respiratory health, which may also encourage outdoor physical activity (19), and reduce risk of premature death (20).

Nearly 1 in 4 adults surveyed reported “no places within a 10-minute walk” as a reason for not walking to near-home destinations. Physical activity is more common in communities that support a mix of businesses and residences near each other (21,22). Communities that have a mix of businesses and housing have become more common in the US (23). However, our findings suggest that opportunities exist to support community walkability. For example, having no places within a 10-minute walk was reported more among Non-Hispanic White adults, adults with higher incomes and education, and those living in the South. Additional research is needed in this area to understand what might be driving this relationship.

About 1 in 5 adults reported “prefer driving or being driven,” “inconvenient,” or “sidewalks missing or poorly maintained” as reasons for not walking to places near home. Though not directly assessed in our study, one plausible explanation for these findings is that multiple transportation options, such as walking, may not be available (24). Furthermore, efforts that support multiple transportation options to everyday destinations can decrease travel distances and improve connectivity for modes such as walking (21). Neighborhoods perceived to have better walkability and safety from vehicles are associated with increased physical activity levels (25). Such factors suggest that interventions to improve neighborhood walkability may help more adults include walking in their daily lives. However, it is also possible that people find walking inconvenient or prefer driving for other reasons. For example, they may want to drive if they must carry groceries or if they have young children with them. To develop interventions to support increased walking, a better understanding of these reasons may be needed.

Our study found differences by sex in the reasons people do not walk to places near home. Across all reasons with differences by sex, more females reported such reasons than males. For example, more females said they preferred driving, which may be influenced by other reasons such as feeling unsafe*.* Safety concerns among females may stem from fears of violence (26). It is unclear from our study whether females are more likely to live in less walkable areas or whether there are other sex-related considerations underlying these differences (eg, females preferring better pedestrian infrastructure, preferring driving to feel safe) (2^6^). Understanding and addressing factors that prevent females from walking to places near home could help with closing the consistently reported sex gap in physical activity (27).

Our findings yielded somewhat surprising results by age group, with younger adults reporting more reasons for not walking near home versus older adults. Two reasons reported for not walking became more common with older age: “cold or icy conditions” and “my physical abilities or fitness.” This aligns with a noted higher risk of falls and fall-related injuries among older versus younger adults and well-documented age-related declines in cardiorespiratory fitness (28). To slow age-related decreases in fitness and strength, older adults are advised to perform at least 150 minutes per week of at least moderate-intensity aerobic activity, plus at least 2 episodes per week of muscle-strengthening activities (28). Furthermore, balance activities are recommended to address concerns over falls and fall-related injuries. The 2023 Physical Activity Guidelines for Americans Midcourse Report contains evidence-based strategies to support physical activity among older adults, including near-home walking (28). National data show transportation walking is less prevalent among older US adults (7). Similarly, younger adults report more positive attitudes toward walking (agreed with the statement “I like walking”) than older adults, and positive attitudes of walking are associated with more walking (29). Yet, we found older adults less likely to report that they did not like walking than younger adults as a reason for not walking near home. More understanding around age-specific preferences for walking and walking environments may help increase transportation walking among older adults and address barriers that impede walking for all age groups.

In our study, adults living in the South more commonly reported several reasons for not walking near home than adults in other US regions. Transportation walking is less common in the South than in other US regions (7), and rates of physical inactivity are highest (30). Our findings suggest that living in the South may present a variety of barriers to outdoor or transportation walking, including hot and humid conditions and a lack of places to walk to that are near home.

### Limitations

Our study has several limitations. First, our sample was recruited from an internet panel, which may introduce sampling bias. Data were weighted to better represent the distribution of the US adult population. Second, having respondents self-report reasons for not walking near home may introduce recall and social desirability biases, but does capture perceptions of the environment, which are important predictors of activity (31). Third, we excluded respondents who had missing data on reasons or who indicated they were unable to walk. Those excluded were more likely than the analytic sample to be females, adults with less education, and adults with lower incomes. Future assessment of mobility barriers among adults who are unable to walk is needed to ensure access to environments for wheelchairs or assisted-walking devices. Fourth, response options did not capture all reasons that prevent people from walking to places near their homes. For respondents who answered none of the above*,* the reasons that prevent them from walking may not be included. Future research can explore additional reasons, including lack of family or social support, to better understand respondents’ reporting none of the above. Fifth, our study was fielded in the summer when some reasons (eg, heat, humidity) may be more salient, which could have affected responses. Sixth, our question referring to “10 minutes from home” may have been interpreted differently by different respondents. Ten minutes from home is based on perceived travel time. Perception of travel time is related to many factors, including one’s primary mode of transportation, the land use in one’s community, and the transportation network (32). Our study intended to explore a wider range of potential reasons that prevent near-home walking than previously reported and can be used to inform future research on specific transportation perceptions. Seventh, this is a cross-sectional study. Longitudinal studies can explore how reasons change over time to better inform interventions that can support active transportation among diverse adults at different time periods. Lastly, our analysis was unable to characterize geographic factors beyond metropolitan statistical area status and region or the places within 10 minutes of the home. 

### Conclusions

Eight of 10 US adults reported environmental, access, or individual reasons for not walking to places within 10 minutes of home. Designing communities to facilitate transportation walking may make it more accessible, convenient, and desirable for adults to add more physical activity to their daily lives. Reasons for not walking may depend on the number and type of options available and whether the destinations are places people want to go to. Future research could pursue data sets with greater availability of geographic variables and objective measures of neighborhood walkability to further study patterns and associations that may better inform interventions to improve transportation walking.
